# Metabolic landscape in cardiac aging: insights into molecular biology and therapeutic implications

**DOI:** 10.1038/s41392-023-01378-8

**Published:** 2023-03-14

**Authors:** Saiyang Xie, Si-Chi Xu, Wei Deng, Qizhu Tang

**Affiliations:** 1 Department of Cardiology, Renmin Hospital of Wuhan University, Hubei Key Laboratory of Metabolic and Chronic Diseases, Cardiovascular Research Institute of Wuhan University, Wuhan, PR China; 2 State Key Laboratory of Complex Severe and Rare Diseases, Department of Cardiology, Peking Union Medical College Hospital, Chinese Academy of Medical Science and Peking Union Medical College, Beijing, PR China; 3 Department of Cardiology, Renmin Hospital of Wuhan University, Department of Cardiology, The Fifth Affiliated Hospital of Xinjiang Medical University, Ürümqi, PR China

**Keywords:** Senescence, Cardiology

## Abstract

Cardiac aging is evident by a reduction in function which subsequently contributes to heart failure. The metabolic microenvironment has been identified as a hallmark of malignancy, but recent studies have shed light on its role in cardiovascular diseases (CVDs). Various metabolic pathways in cardiomyocytes and noncardiomyocytes determine cellular senescence in the aging heart. Metabolic alteration is a common process throughout cardiac degeneration. Importantly, the involvement of cellular senescence in cardiac injuries, including heart failure and myocardial ischemia and infarction, has been reported. However, metabolic complexity among human aging hearts hinders the development of strategies that targets metabolic susceptibility. Advances over the past decade have linked cellular senescence and function with their metabolic reprogramming pathway in cardiac aging, including autophagy, oxidative stress, epigenetic modifications, chronic inflammation, and myocyte systolic phenotype regulation. In addition, metabolic status is involved in crucial aspects of myocardial biology, from fibrosis to hypertrophy and chronic inflammation. However, further elucidation of the metabolism involvement in cardiac degeneration is still needed. Thus, deciphering the mechanisms underlying how metabolic reprogramming impacts cardiac aging is thought to contribute to the novel interventions to protect or even restore cardiac function in aging hearts. Here, we summarize emerging concepts about metabolic landscapes of cardiac aging, with specific focuses on why metabolic profile alters during cardiac degeneration and how we could utilize the current knowledge to improve the management of cardiac aging.

## Introduction

In the past few decades, improved pharmacological and surgical treatments have contributed to increased life expectancy and an aging population in certain industrialized countries.^1^ In addition, the proportion of the retired population is rapidly increasing; retirees are much more likely to slide into sedentary lifestyles, which further accelerates cardiac aging.^2–5^ The incidence of age-associated disorders, especially cardiovascular diseases (CVDs), has increased dramatically; in elderly individuals, these age-related disorders correlate with high hospitalization, increased mortality rates, as well as elevated cost.^4,5^ Even without disease, aging is accompanied with functional decline of multiple organs. It is characterized by unique histological and biochemical features, including oxidative stress, protein misfolding, cell death, and mitochondrial abnormalities.^6,7^ Similar to other group of organs, the heart function declines gradually with age, which is evident by the reduced pump function and myocardial compliance resulting from increased afterload and insufficient coronary perfusion/oxygenation.^8–10^ Conventional wisdom suggests that the aging heart manifests increased left ventricular mass, while considerable evidence from autopsies and magnetic resonance imaging demonstrate that cardiac mass tends to be decreased in older adults and remain the same in women in the absence of hypertension.^11,12^ In line with the reduced performance of other organ systems, the age-related cardiac decline dramatically accelerates after 50 years of age.^10^

In elderly individuals, the metabolic landscape during cellular senescence can increase the risk of cardiac function dysregulation and cardiac repair dysfunction.^13^ For example, the stroke volume of the aging heart is mildly increased during moderate aerobic exercise. This is partly due to the strength of the Frank-Starling mechanism (cardiac preload), in which the metabolic demand of the work rate is substantially enhanced.^14,15^ In addition, a recent study involving metabolic profiling of serum and urine in healthy subjects suggested that aging induces impaired catabolism in glycoproteins, amino acids, and several lipids, among which cumulative metabolites contribute to the cardiac aging process.^16^ Importantly, accumulated evidence from multiomics exploration has highlighted the complex alterations of metabolic status during aging. In this regard, we review and outline the recent advancements of the mechanisms and therapeutic implications of the metabolic landscape in aging hearts since this information influences the progression of the cardiac dysfunction and lifespan.

## Distinct substrate metabolism during cardiac aging

**Fig. 1:**
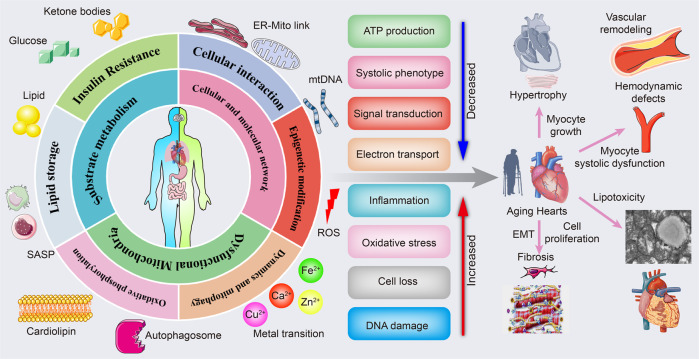
The link between cardiac aging and metabolic pathology. Metabolic drivers of cardiac aging. Mitochondrial substrates metabolic disturbance including lipid storage and insulin resistance; dysfunctional mitochondria with impaired oxidative phosphorylation, mitochondria dynamics, and mitophagy; cellular and molecular network. All of which drive reduced ATP production, systolic phenotype, signal transduction, and electron transport, along with increased inflammation, oxidative stress, cell death, and DNA damage. Finally, various pathological alterations, including myocyte growth-induced cardiac hypertrophy, endothelium–mesenchymal transition and cell proliferation-mediated cardiac fibrosis, lipid deposition-induced comparative cardiac lipotoxicity, and insufficient energy-mediated myocyte systolic dysfunction and hemodynamic disorder, contribute to cardiac aging, thus the failing heart. The online resource inside this figure was quoted or modified from Servier Medical Art

Aging hearts are accompanied by several pathological changes in metabolism (Fig. 1). Below, we outline the major alterations in metabolism and their involvement in developing cardiac aging.

### Glucose metabolism

Unlike the adult heart, the aging heart experiences proportionally reduced myocardial lipid catabolism. In addition, anaerobic glycolysis, instead of glucose oxidation, gradually dominates the energy source in the aging heart,^17^ which is in line with pathophysiological changes such as cardiac hypertrophy and impaired contractile function.^18^ Furthermore, aging is associated with obesity-independent insulin resistance, and involves disrupted mitochondrial structure and dysregulated cellular insulin action.^19^ Notably, circulating glucose is substantially increased due to the compromised ability of glucose transporters (GLUTs) to transfer glucose during aging, thus causing elevated fasting blood insulin and glucose levels.^20^ Emerging evidence has shown that culturing cells under hyperglycemia or high insulin conditions accelerates cellular senescent phenotype.^21,22^ Consistently, age-dependent insulin resistance and glucose intolerance may result in diabetes, CVDs, and stroke and are associated with poor cardiac function during aging.^23^ Moreover, positron emission tomography imaging of radio-tagged glucose absorption in the aging heart demonstrated insulin resistance and damaged glucose shuttling, as well as the proportional reduction in FA oxidation and lipid accumulation.^24,25^ In energy provision, glycolysis is unlikely to compensate for impaired glucose oxidation and FA utilization, similar to cardiac ischemia-induced heart failure. Hence, anaerobic glycolysis synergizes with reduced FA utilization, resulting in an irreversible and persistent energy deficit and aberrant cardiac contraction. In addition, the enhanced pentose phosphate pathway impedes glucose utilization in the aging heart.^26^ Simultaneously, activation of the pentose phosphate pathway may also induce impaired FA oxidation and higher lipofuscin accumulation in cardiomyocytes, leading to cardio-lipotoxicity.^27^ However, the mechanism behind this remains unclear. Accordingly, the carboxylation of pyruvate to malate without acetyl-CoA production might partly account for the increased glycolytic flux.^28,29^ The latter is also known as an anaplerotic reaction and partly counteracts impaired pyruvate oxidation even though less energy is produced than that with the unabridged Krebs cycle in mitochondria.^30^ It seems that anaplerotic reactions partly compensate for insufficient fueling and prevent accumulation of pyruvate in the heart.

Cardiac aging molecular mechanisms are sophisticated, making it impossible to utilize unifying model in deciphering the hyperglycemia-associated cell senescence. Reduction in growth hormone (GH) and insulin-like growth factor (IGF) and increased insulin responsiveness correlate with the prolonged life and an apparent reduction in the aging process.^31–33^ IGFs, insulin receptors (INSR), and insulin receptor substrate-1 (IRS-1) could regulate insulin resistance as well as contribute to metabolic syndrome (Fig. 5). With regard to cardiac aging, the interaction of insulin-like growth factor-1 (IGF-1) with IGF-1 receptor (IGF-1R) accelerates myocardial pathologies in cardiac aging and longevity in mammals.^34^ Consistently, forced cardiac expression of the IGF receptor in Drosophila promotes cardiac aging.^35^ Cardiomyocyte-specific absence of IGF-1R impedes the initiation of senescence-related myocardiopathy. Contradictorily, some studies revealed that endogenous IGF-IR pathway diminishes the age-correlated diastolic dysfunction.^36,37^ In IGF-1 overexpressing mice, dysregulated diastolic and contractile activity of aging hearts was improved, and cardiac aging was delayed by preservation of cardiac SERCA expression and activity.^38,39^ Conversely, another study revealed that pharmacological targeting of cardiac IGF-1 pathway could provide a undiscovered strategy for cardiac health and lifespan extension.^40^ These results emphasize the regulatory function of IGF-1/IGF-1 receptor during aging progression, leading to a difficult discrimination between the cardiac and systemic consequences of IGF-1. To solve this puzzle, plasma IGF-1 deficiency^41,42^ and controlled inhibition of IGF-1R in cardiomyocytes^37^ were subsequently investigated; the beneficial effects of forced expression of IGF-1 in heart tissues may be delineated by elevated blood IGF-1 concentration, and IGF-1 promoted the function restoration of the ischemic heart.^43^ Hence, cardiac and systemic IGF-1 had distinct effects on cardiac aging.

**Fig. 2:**
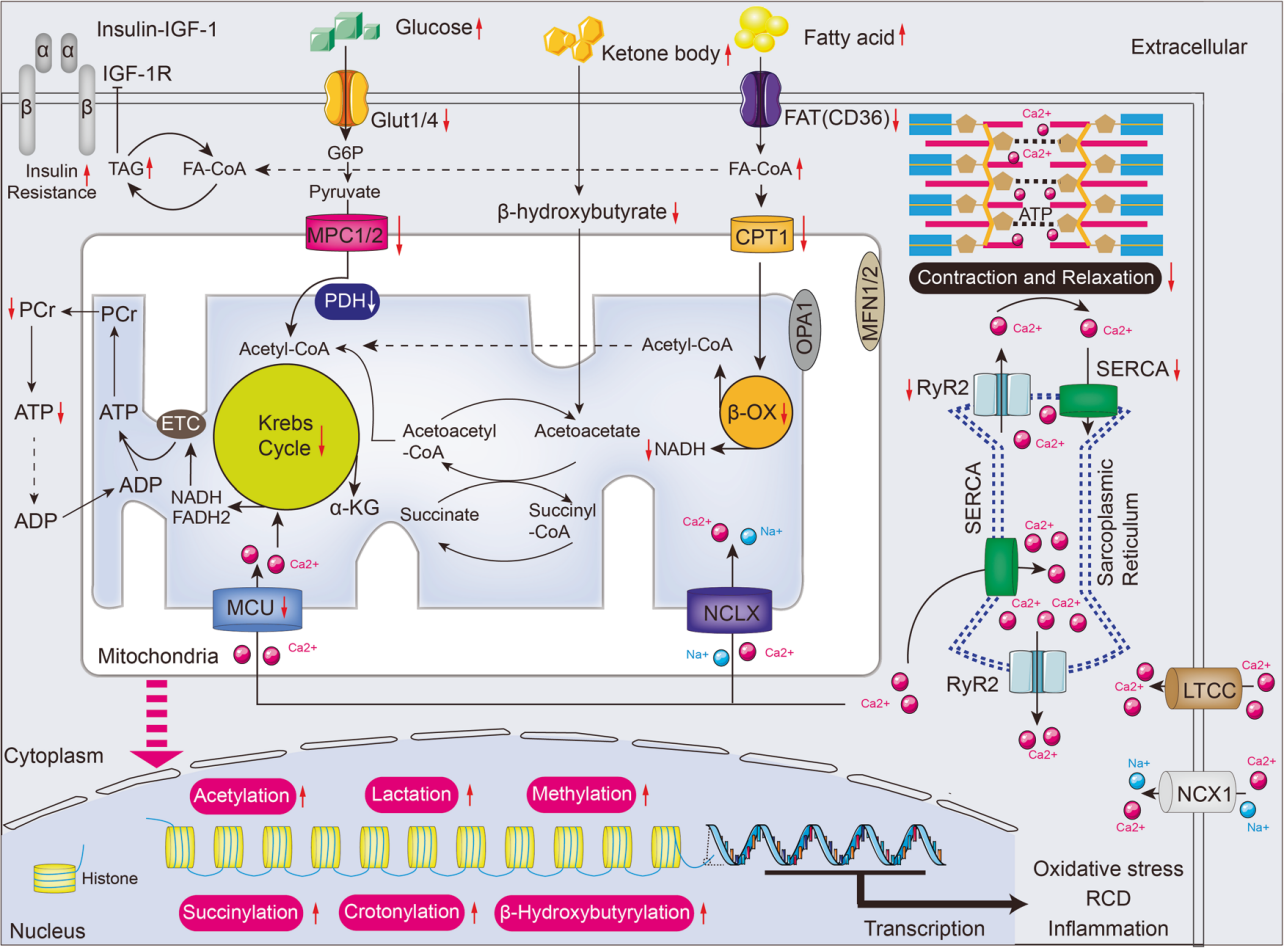
Metabolic substates utilization and excitation-contraction coupling in the aged heart. In the aging heart, myocardial lipids catabolism and glucose oxidation are reduced due to insulin resistance, but glycolysis dominates the energy source. Also, the relative contribution of ketone body utilization to ATP production is enhanced. The mismatch between FA oxidation and uptake results in the accumulation of toxic lipid intermediates, leading to impairment of ATP production and formation of high-energy phosphates. Furthermore, several metabolic intermediates interfere with metabolism and course oxidative stress, RCD, and inflammation by post-translational modifications and serving as chromatin-modifying enzymes in cardiac aging. Krebs cycle and excitation-contraction coupling are stimulated by Ca^2+^ that mainly sources from sarcoplasmic reticulum (SR), while the transport of Ca^2+^ is disturbed in the aging heart. Red arrows indicate alterations that occur in the aging heart (see text for details). α-KG α-ketoglutarate, ADP adenosine diphosphate, ATP adenosine triphosphate, β-OX fatty acid β-oxidation, CPT1 carnitine O-palmitoyltransferase 1, ETC electron transport chain, FADH_2_ reduced flavin adenine dinucleotide, FAT fatty acid translocase (also known as CD36), FA-CoA fatty acyl-CoA ester, Glut4 glucose transporter 4 (also known as SLC2A4), G6P glucose-6-phosphate, IGF-1 Insulin-like growth factor 1, LTCC L-type Ca^2+^ channel, MFN1/2 Mitofusin1/2, MPC1/2 mitochondrial pyruvate carrier1/2, MCU mitochondrial Ca^2+^ uniporter protein, NADH nicotinamide adenine dinucleotide, NCLX mitochondrial Na^+^/Ca^2+^ exchanger protein, NCX1 Na^+^/Ca^2+^ exchanger 1, OPA1 dynamin-like guanosine triphosphatase, PCr phosphocreatine, PDH Pyruvate dehydrogenase, RCD regulated cell death, RYR2 ryanodine receptor 2, SERCA sarcoplasmic/endoplasmic reticulum Ca^2+^ ATPase, TAG triacylglycerol. The online resource inside this figure was quoted or modified from Servier Medical Art

As the top two abundant glucose transporters in the heart, glucose transporter-1 (GLUT1) localizes in the sarcolemma and regulates cardiac glucose shuttling at basal state, which is dominant in resting cardiomyocytes;^44^ however, glucose transporter-4 (GLUT4), the primary variant making up about 70% of all glucose transporters, localizes in a particular cellular compartment and shuttles into the plasma membrane upon cardiomyocyte contraction and insulin insult.^44^ Of note, GLUT4 (also known as SLC2A4) can be impaired during aging, reducing glucose uptake and utilization in cardiomyocytes^45^ (Fig. 2). Substantial studies have suggested that advanced glucose end products (AGEs) also correlate with cardiomyopathy, especially in the heart of older individuals with diabetes.^46–48^ AGEs accumulate in the myocardial interstitium, resulting in excessive cross-linking of ECM proteins, which leads to cardiac muscle rigidity and diastolic dysfunction.^49–51^ Furthermore, AGEs specifically interact with the receptor for advanced glycation end products (RAGE) to activate NF-κB nuclear translocation, which stimulates excessive ROS production and increases the expression of p21 or p16 in the myocardium.^52–55^ However, how circular AGEs are taken up by cardiomyocytes and removed from cardiomyocytes is poorly understood. More importantly, despite the effectiveness of the short-term intervention targeting of the AGE-RAGE axis,^53^ chronic AGE inhibition was not considered. More research is therefore needed to fully elucidate circular AGE transportation and the AGEs in people with aging hearts.

Besides cardiomyocytes, crosstalking between cardiac fibroblasts and cardiomyocytes inhibits glucose metabolism enzyme activity and lactate transporter expression, possibly due to the fibroblast growth factor 21 (FGF21)-adiponectin pathway during aging-related heart failure.^56,57^ More importantly, defective glucose metabolism in the aging heart is correlated with immune activation in noncardiomyocytes. Notably, elevated insulin promotes T-cell activation during aging, with a subsequent increase in insulin receptor and glycolytic enzymes, both of which are required for adaptive immunity.^58,59^ In brief, upregulation of the insulin receptor contributes to polyclonal activation of CD4^+^ and CD8^+^ T lymphocytes and, subsequently, numerous secretory proinflammatory cytokines, including IFN-γ, TNF, and IL-17.^58^ Importantly, increased insulin concentrations also impede regulatory T cells (Tregs) in antagonizing inflammation, which serves as the trigger of cardiac aging.^60^ These data suggest that the T-cell-mediated immune response might be an important trigger for local inflammation in cardiac aging. A prior publication revealed that restored metabolism profiling in myeloid cells counteracts cognitive impairment in the aging central nervous system (CNS), in which aged brain tissues are susceptible to inflammation induced by aging macrophages and microglia.^61^ Likewise, chronic inflammation in the aging heart is triggered largely by myeloid-derived macrophages, whereas how hyperglycemia impacts cardiac macrophages during aging is not known. Numerous studies have also demonstrated that hyperglycemia stimulates cellular senescence in endothelial cells partly due to reduced arginase 1 (ARG1) expression and nitric oxide synthesis (NOS),^62,63^ a crucial product for vessel growth and angiogenesis. In addition, elevation of glucose concentrations induced senescence in cardiac fibroblasts (CFs) by promoting telomere shortening,^64^ which may be caused by the senescence-associated secretory phenotype (SASP)^65^ of other cell types due to the hyposensitivity of isolated CFs in response to hyperglycemia. Therefore, the involvement of glucose in regulating senescence of various types of cells should be further determined.

### Lipid metabolism in the aging heart

Dyslipidemia, including high cholesterol, hypertriglyceridemia, and elevated low-density lipoproteins (LDLs), triggers thrombosis and increases the risk of CVDs.^66^ Inside the aging heart, dysregulated cardiac function is linked with reduced oxidation of FA. This is indicated by the accumulation of free fatty acids (FFAs) and lipid-laden cells in heart tissues,^30,67^ consistent with pathological cardiac hypertrophy.^18^ In contrast, FA delivery into cardiomyocytes is promoted in individuals with advanced aging conditions. Therefore, the imbalance between FA uptake and utilization leads to excess in the intracellular lipids, triggering toxic lipid species (including ceramide and diacylglycerol) and eventually lipotoxicity during cardiac aging.^68^ Besides aging directly impeding FA oxidation, increased insulin contributes to the inactivation of FA oxidation by restricting the activity of major rate-limiting enzymes in cardiomyocytes.^69^ Worse of all, the intracellular accumulation of lipids further causes posttranslational modifications of several components that regulate insulin production. Consequently, insulin resistance is accelerated, and cellular senescence is further promoted.^70,71^ These lines of evidence indicate that abnormal cardiac lipid metabolism promotes cardiac aging (Fig. 2). The mismatch between lipid uptake and oxidation drives cardio-lipotoxicity and partly accounts for insulin resistance during aging.

Increased cardiac CD36 supports FA transport during aging and sustains enhanced cardiac lipid content.^72^ Accordingly, aged CD36-depleted mice showed reduced lipid accumulation in heart tissues, and improved ATP production and cardiac dysfunction.^73^ Conversely, a high-fat diet (HFD) and CD36 overexpression promote FA uptake and higher cardiac lipid content during aging.^74^ Moreover, peroxisome proliferator-activated receptor-α (PPAR-α) signaling and peroxisome proliferator-activated receptor-γ coactivators (PGC1s) are overtly suppressed in aging-induced cardiac dysfunction,^75,76^ while aging promotes PPAR-gamma activation,^77^ all of which dysregulate FA mitochondria shuttling and downregulates its oxidative enzymes in cardiomyocytes.^78^ In addition, ceramide, a lipid metabolite, has been reported to promote senescence by inducing a reduction in cardiolipin content and mitochondria dysfunction in cardiomyocytes.^79^ Recently, it has been suggested that enhanced ceramide catabolism restrains cardiac lipotoxicity in type 2 diabetic mice,^80^ which further supports the adverse cardiac influence of ceramide, but the molecular machinery is less understood. Additional studies indicate that the release of proinflammatory factors of the SASP, sourced from lipid metabolites, was dramatically increased in the aging heart, whereas monounsaturated fatty acids (MUFAs), functioning as a lipid-lowering metabolite, were substantially reduced.^81^ Moreover, accumulating evidence suggests that exogenous lipids accumulate and are incorporated into triacyclglycerols to form numerous lipid droplets in the aging heart.^82^ In line with glucose metabolism, lipid metabolism was also verified to modulate T-cell activity in cardiac aging.^83^ It has been confirmed that macrophage FA oxidation can suppress atherosclerosis,^84^ but the impacts of macrophage lipid metabolism on cardiac aging are not fully explained. In addition to lipid droplets, fatty acids are also converted to oxylipins, among which increases in proinflammatory prostaglandin E2 (PGE2),^85^ prostaglandin synthase 2 (PTGS2 or COX-2),^86,87^ thromboxanes (TXs),^88^ and leukotrienes (LTs)^89^ and decreases in anti-inflammatory lipoxin A4,^90^ resolvins,^91^ protectins, and maresins are associated with cellular senescence.^92^ Moreover, the enhancement of cytokines sourced from lipid metabolism recruits and promotes cardiac fibroblast proliferation, thereby inducing wall stiffness and diastolic dysfunction in the aging heart. These data suggest crucial impacts of inflammation and lipid metabolism on cardiac aging, and various aspects regarding lipid metabolism remain mysterious in the context of cardiac aging.

### Ketone bodies: a compensatory fuel

Ketone bodies, comprising beta-hydroxybutyrate, acetoacetate, and acetone,^93^ are derived from FA oxidation and serve as primary body energy sources during fasting and ketogenic diet (KD) in physiological homeostasis.^94^ In previous studies, ketone body oxidation-related enzymes and intermediates derived from its metabolism were increased in both preclinical models and heart failure patients,^95,96^ implying that ketone bodies serve as a critical optional energy in heart failure^97^ (Fig. 2). Given the impaired FA oxidation and glucose utilization in aging, ketone bodies could be an essential substrate that alleviates aging-related cardiac dysfunction and serves as a compensatory fuel. Expectedly, similar to the aging brain that metabolizes 3-beta-hydroxybutyrate (3HB) and acetoacetate (AcAc) ketone bodies,^98^ increased ketone body flux indeed improves cardiac dysfunction in the aging heart,^99^ by which the heart compensates for contractile function and manifests cardiac hypertrophy with aging. Besides being the source of energy, it metabolites β-hydroxybutyrate, a histone deacetylases antagonist, induces cell proliferation and inhibits inflammation;^100^ thus, β-hydroxybutyrate may serve as a blocker of aging-related inflammation. Hence, the ketogenic diet is beneficial for aging-related cardiovascular complications.

The high level of ketone body flux during cardiac aging is necessary to support energy metabolism and increase cardiac metabolic efficiency. In some way, beta-hydroxybutyrate catabolism provides higher ATP production [2.55 vs. 2.33 in ATP production: oxygen consumption ratio (P:O)] than FA palmitate.^101^ Beta-hydroxybutyrate is regarded as an adjunctive nutritional therapy for aging.^102^ As a mitochondrial enzyme responsible for the liver-independent ketone body metabolism, cardiac succinyl-CoA-3-oxoacid CoA transferase (SCOT) presents substantially increased activity in aged animals.^103^ In mice, the absence of SCOT increased vulnerability to ketosis and reduced plasma levels of glucose and lactate.^104^ In addition, several studies demonstrated the metabolic effects of ketone bodies, which exert antioxidant effects by promoting the proportion of reduced and oxidized glutathione, thus directly counteracting oxidative stress and removing oxygen radicals.^105,106^ Moreover, increased ketone body flux benefits mitochondrial restoration by causing impaired mitochondria removal through Parkin-mediated mitophagy during cardiac aging.^107^ Although ketone body flux induces impaired mitochondria removal, the crosstalk between mitophagy and FA oxidation remains exclusive in the context of cardiac aging. Ketogenic diet is reported to decrease midlife mortality and improve memory; simultaneously, the cyclic KD maintained a cardiac phenotype that resemble to young mice in aging mice.^108,109^ Moreover, a KD inhibited longevity-associated signaling of insulin and mTOR pathway. Notably, it triggered PPAR alpha, a leading factor that governs the transcription of genes for ketogenesis and mitochondrial homeostasis^108,109^ (Fig. 5). Notably, this study focused only on the short-term impacts of KD, whereas its long-term influences on age-related cardiomyopathy are poorly understood. Moreover, the link between diabetes mellitus (DM) and aging is involved in ketogenesis, which has attracted more attention,^110,111^ and enhanced cardiac aging in the context of DM makes it intriguing research. In addition, the effects of ketone bodies on cardiac fibroblasts, endothelial cells, and immune cells are not known.

### Acetyl-CoA and epigenetic intermediates

Acetyl-coenzyme A (acetyl-CoA) is a crucial cofactor in regulating metabolism. Being the end metabolite of FA oxidation and glycolysis, acetyl-CoA fuels the Krebs cycle and synthesizes ketone bodies. In addition, fluctuations in acetyl-CoA concentration are reported to be involved in alterations in histone modification that loosen or promote their interaction with DNA, therefore regulating gene expression.^18,112^ Both ATP-citrate lyase (ACLY) and acetyl-CoA synthetase 2 (ACSS2) are responsible for acetyl-CoA synthesis.^113,114^ The incapability of ACLY results in the reduced nuclear production of citrate-mediated acetyl-CoA, which simultaneously suppresses the acetyl-transferase activity of p300 and results in increased autophagy, which prevents aging outcomes.^115^ Moreover, shuttling of the pyruvate dehydrogenase complex (PDC) from mitochondria to the nucleus triggers the production of nuclear acetyl-CoA for histone modification and epigenetic regulation,^116^ which may serve as a trigger for aging progression. In addition, acyl-coenzyme A (CoA)-binding protein (ACBP), or diazepam-binding inhibitor (DBI), which reduces cardiac fibrosis, is a regulatory factor for autophagy,^117^ supporting the contention that ACBP induces cardioprotection, probably in cardiac aging. In addition, cytoplasmic ACSS2 hinders autophagy from causing aging, inducing AMPK-mediated ACSS2 nuclear translocation, which functions more drastically than cytoplasmic ACSS2 in aging outcomes. Specifically, nuclear ACSS2 accumulation upregulated the transcriptional capacity of HATs, including CREB-binding protein (CBP) and p300/CBP-associated factor (PCAF),^118,119^ which serve as enhancer elements to stimulate the transcription of cardioprotective genes and facilitate lysosomal biogenesis and autophagy during cardiac aging.^112^ The distinct location of ACSS2 results in opposite aging outcomes, implying the functional diversity of acetyl-CoA in control of cardiac aging. Consistent with acetyl-CoA, acetate is also regarded as the substrate of ACSS2 to revert the aging phenotype in cultured stem cells.^120^ Hence, acetyl-CoA governs metabolic hemostasis by operating concurrently as a metabolic product and a secondary messenger during cardiac aging. Several forms of modifications, including methylation, lysine beta-hydroxybutyrylation (Kbhb), and succinylation (Fig. 2), have been found to be involved in CVDs.^121^ Nevertheless, the impacts of histone modification on cardiac aging and longevity remain to be fully studied. Most recently, other metabolites and associated posttranslational modifications (PTMs), such as malonylation,^122,123^ lactylation,^124,125^ crotonylation,^126^ and glutarylation,^127^ have been verified in cardiac metabolism. Therefore, studies with emphasis on the influence of metabolite-related modifications on controlling autophagy and transcription, which are important drivers of organismal aging, are needed.

### Energy fuel, Ca^2+^, and myocyte systolic phenotype

In cardiac muscle, excitation-contraction coupling (ECC) directly connects membrane depolarization with contraction, and most energy obtained from OXPHOS in mitochondria is burned off to fuel the incessant myocyte systolic phenotype.^128^. Cardiac ECC requires enormous amount of cellular energy; the primary energy users are myosin ATPase, the ion exchanger ATPase, and SERCA.^129^ Of note, energy consumption and Ca^2+^ transporting rates are associated with post-translational modifications. As an example, both the phosphorylation of SERCA^130^ and RyR2,^131^ and the deacetylation of SERCA,^132^ enhance Ca^2+^ transporting rate and cardiac contractility. The heart is enriched with metabolic components that serve OXPHOS in mitochondria to fulfill its energy requirements. However, distinguished alterations, i.e., some components are decreased (Cav1.2, Cav1.3, HCN4, and RYR2), while others (NCX and SERCA densities and proteins) are increased in the aging rat heart.^133^ In addition, ROS-induced SERCA oxidation at Cys674 results in SERCA inactivation and myocyte relaxation impairment in the senescent heart.^134^ Although the link between Ca^2+^ and myocyte systolic phenotype has been verified, connections between impaired metabolism, calcium homeostasis, and myocyte systolic phenotype during cardiac aging are poorly understood.

## Mitochondrial impairment during cardiac aging

### Mitochondrial morphology

As a factory with high energy demands, the heart is very rich in mitochondria, which generate approximately 90% of ATP to maintain pump function in the heart. Depending on the location, cardiac mitochondria are classified into subsarcolemmal mitochondria (SSM) and interfibrillar mitochondria (IFM) that have different activities.^135^ Specifically, defective IFM results in cardiac aging, and the decrement of IFM is associated with aging-related cardiac dysfunction,^136^ while that of SSM is unchanged during aging. These data hint a crucial role of IFM in cardiac aging progression. Using transmission electron microscopy (TEM), mitochondrial morphology was evaluated on cardiomyocytes obtained from aging rats. The heart muscle section area analysis revealed that the inner mitochondrial membrane (IMM) per unit volume of mitochondria was dramatically reduced in aging.^137^ However, in prior studies, no age-related alterations in cristae morphology were observed in heart tissues of aging rats by TEM.^138,139^ Moreover, impaired IFM renewal and decreased elimination of IFM led to the accumulation of abnormal IFM bearing elevated ROS amount in monocytes, all of which accelerated oxidative stress and the aging phenotype.^140,141^ In some ways, the species gap of rats and slightly different fixation may have contributed to this distinctive outcome. In addition, several clinical and preclinical studies reported that the aging heart possesses swelling mitochondria characterized by broken inner membrane cristae.^135,142^ Together, aging impairs mitochondrial integrity with defective IFM in the heart, which may be the earliest alteration in morphology prior to cardiac hypertrophy and fibrosis.

Cardiolipin, a major diphosphatidylglycerol lipid of the IMM,^143^ is also essential for mitochondrial function. Cardiolipin levels decline substantially in cardiac aging,^144^ and aging results in decreased membrane fluidity in the inner membrane, which further regulates electron transport. Moreover, aging drives ROS enrichment in cardiolipin, and cardiolipin oxidation by cytochrome c results in age-enhanced oxidative damage to mitochondria.^145^ Beyond that, cardiolipin may serve as the susceptor to perceive senescence stress and induce signal transduction in mitochondria, while whether cardiolipin participates in lipid metabolism inside mitochondria and the potential mechanism remains unclear. In the future, mitochondrial morphometric analysis during cardiac aging should be conducted in more species, particularly primates.

Mitochondria undergo the processes of biogenesis, dynamics (fusion/fission), and mitophagy. These events not only are crucial for its function but also likely play a role during aging.^146^ Biogenesis of mitochondria is an elaborated process to regenerate mitochondria from existing ones.^147^ In addition, mitochondrial biogenesis is affected by aging, and PGC-1α is reported to govern this event and serves as an attractive therapeutic target.^148^ Specifically, PGC1 stimulates the expression of uncoupling protein 2 (UCP-2) and the nuclear respiratory factors (NRFs), both of which contribute to mitochondrial DNA replication/transcription.^148^ However, the link between the phenotypes observed with PGC1 enrichment in the nucleus and increased deoxyribonucleoside triphosphate (dNTP) synthesis is unclear. Compared to the neonatal heart, PGC-1α activation in aging cardiomyocytes leads to lower mitochondrial biogenesis, causing reduced specific proliferation within the myocytes.^149^ In contrast, PGC-1α impedes the senescent phenotype in vascular smooth muscle cells (VSMCs) by restoring mitochondrial biogenesis and p62-mediated mitophagy.^150^ Of note, reduced length of telomere and mitochondrial defection are common initiators of aging,^151^ in which PGC-1α/β establishes the connection.^152–154^ Overexpression of PGC-1α is reported to restore muscle aging by disturbing p53-induced DNA damage and telomere dysfunction.^155^ Consistently, an emerging study indicated that telomere shortening antagonized PGC-1β-mediated mitochondrial biogenesis and ROS production to induce the aging process.^156^ Therefore, abnormal PGC-1α/β expression is also involved in the link between reduced length of telomere and mitochondrial defectiveness. However, decreased length of telomere is associated with mitochondrial function and metabolism during early aging via uncertain mechanisms. In addition to PGC-1α, multiple regulators, such as sirtuin, AMPK,^157^ Nrf2^158^, and lncRNAs,^159^ have been suggested to control mitochondrial biogenesis during aging. However, how they impact cardiac aging deserves further investigation.

**Fig. 3:**
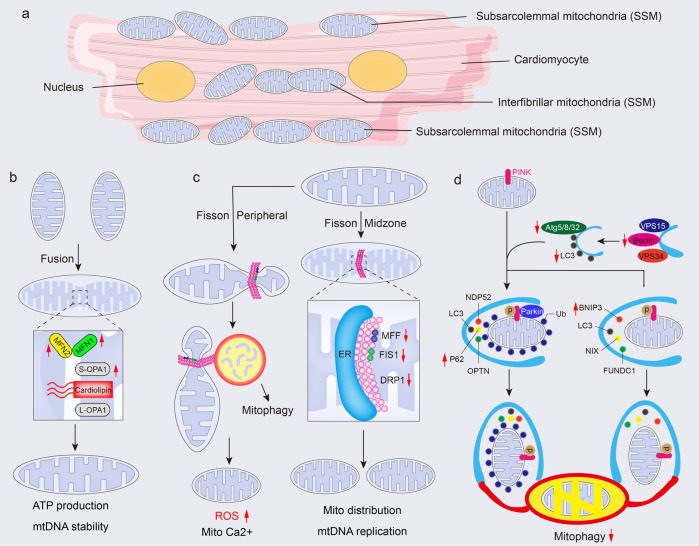
Mitochondrial dynamics and mitophagy in the aged heart. **a** A schematic of a cardiomyocyte to highlight the location of subsarcolemmal (SSM) and interfibrillar mitochondria (IFM). **b** Mitofusins (MFN1 and MFN2) in the OMM, belongs to proteins of the dynamin-related family of large GTPases, synergizing with OPA1 in the IMM to regulate mitochondria fusion. In the aging heart, long OPA1 (L-OPA1) is cleaved to generate the short form of OPA1 (S-OPA1), and the latter cooperates with cardiolipin to promote the fusion of mitochondria, by which compensates the ATP production and maintains mtDNA stability. **c** Fission is predominantly orchestrated by the DRP1. DRP1 binds to OMM receptors MFF and mitochondrial FIS1, promoting the midzone fission to course mitochondria distribution with mtDNA replication. Also, DRP1 induces peripheral fission and enables damaged material to be destined for mitophagy. The latter results in higher ROS generation and enhanced mitochondria Ca^2+^. **d** As a serine/threonine-protein kinase, PINK1 serves as the sensor that detects impaired mitochondria and leads to the proteolytic cleavage of PINK1 by mitochondrial proteases. Uncleaved PINK1 plays a role in activating parkin through direct phosphorylation of the parkin Ub-like (UBL) domain, as well as phosphorylation of ubiquitin. This, in turn, recruits autophagy receptors such as p62, OPTN, and NDP52, promoting the recruitment of LC3 and subsequent engulfment of damaged mitochondria by autophagosomes. Also, autophagy receptors such as BNIP3, NIX, and FUNDC1 regulate ubiquitin-independent mitophagy by recruiting LC3 and facilitating the engulfment of damaged mitochondria by autophagosomes. Red arrows indicate alterations that occur in the aging heart (see text for details). Atg autophagy-related protein, ATP adenosine triphosphate, BNIP3 bcl-2 19-kDa interacting protein 3, DRP1 dynamin-related protein 1, ER endoplasmic reticulum, FIS1 fission 1 protein, FUNDC1 FUN14 domain containing 1, IMM inner mitochondrial membrane, MFF mitochondrial fission factor, MFN mitofusin, mtDNA mitochondria DNA, NDP52 Nuclear dot protein 52 kDa, OMM outer mitochondrial membrane, OPA1 optic atrophy 1, OPTN optineurin; ROS reactive oxygen species, VPS (also known as PI3KR4) phosphoinositide 3-kinase regulatory subunit 4. The online resource inside this figure was quoted or modified from Servier Medical Art

The aging heart also presents abnormal mitochondrial dynamics with aberrant mitochondrial fission and fusion to regulate cardiac energetic homeostasis under oxidation; both are suggested to mediate the aging process.^160^ Substantial studies indicate that the promotion of fission or blockade of fusion of mitochondria impedes cellular senescence.^161,162^ This notion is supported by animal models or isolated cells with proper regulation of mitochondrial fission protein 1 (Fis1) or mitofusin-1 content.^163,164^ In detail, the skeletal muscle obtained from aging mice shows higher mitofusin-1 and mitofusin-2 and reduced Fis1 content, favoring the notion that fusion supports cardioprotection against oxidation.^165^ Moreover, cardiac aging is characterized by insulin resistance, accompanied by FA and ROS accumulation in cardiomyocytes, which contributes to the mitochondrial fusion induced by the mismatching between protein-optic-atrophy 1 (OPA1) and dynamin-1-like protein (DRP1).^166^ OPA1 enables precise control in mitochondrial fusion, mitochondrial DNA preservation, energy exchange, and cristae integrity (Fig. 3a). Conversely, DRP1 serves as a pro-fission protein and controls mitochondrial shape (Fig. 3b).^167^ As expected, the equation of fission and fusion preserves mitochondrial dynamics and respiration to oppose cellular pathology, such as senescence. Of note, mitochondrial dynamics proteins also serve as the link between ER and mitochondria,^168^ and the crosstalk of both in cardiac aging requires further exploration. Generally, defective mitochondria are eliminated by mitophagy to prevent oxidative stress during aging, in which there is potential for membrane depolarization to stimulate mitophagy in a Parkin-dependent manner.^169^ Hence, targeting mitochondrial dynamics and mitophagy (discussed blow) could serve a potential management of age-related cardiac changes.

### Mitophagy: an intrinsic scavenger

Mitophagy facilitates the disposal of damaged or excess mitochondria by autophagy specifically targeted to mitochondria. It is a process that specifically targets and degrades whole mitochondria for their removal.^170^ In contrast, the buildup of impaired mitochondria and presence of cellular dysregulation, inducing aging and age-predisposed cardiac dysfunction,^171^ can result from defective mitophagy.^172^ Inactivation of autophagy accelerates the aging-related aggregation of misfolded proteins,^173^ dysfunctional mitochondria^174^ and subsequent ROS generation^175^ in cardiomyocytes, disrupting the cellular environment and promoting the aging-associated cardiac phenotype. Different autophagy-related genes differentially regulate the various stages of autophagy. It has been proposed that Atg5 transgenic mice present boosted autophagic potential in the myocardium, contributing to health benefits such as decreased cardiac fibrosis and increased lifespans when compared with aged-matched control mice.^176^ Consistently, Becn1^F121A/F121A^ knock-in mice, generated by blocked interaction of Bcl-2 with Beclin1, presented enhanced autophagy and reduced cardiac hypertrophy and interstitial fibrosis.^177,178^ Conversely, impeding cardiac autophagy contributes to advanced aging of heart accompanied by cardiac hypertrophy and the accumulation of dysfunctional mitochondria.^179^ Accordingly, increasing evidence indicates that mitophagy is absent during cardiac aging, leading to oxidized and damaged lipofuscin, which serves as a producer of oxygen radicals and further aggravates mitochondrial damage in aged hearts.^180^ In terms of molecular metabolism, PTEN-induced putative kinase 1 (PINK1)-Parkin-mitofusin2 (Mfn2) labels dysfunctional mitochondria, and autophagosomes are recruited in an LC3-receptor-dependent manner to engulf the targeted mitochondria for removal (Fig. 3c).^181^ A prior study found that the ablation of Parkin in aging mice led to abnormal mitochondrial accumulation in cardiomyocytes,^182^ indicating the crucial role of Parkin-mediated mitophagy during cardiac aging. Interestingly, Parkin-deleted mice showed an boosted aging potential and accumulated abnormal mitochondria in the aging heart,^182^ while forced expression of Parkin in the heart improved mitochondrial health and slowed down aging process in the heart,^183^ in which the cardioprotective effects of Parkin were dependent on mitochondrial DNA.^184^ More importantly, long-term Parkin overexpression or high Parkin expression results in cardiac fibrosis,^185^ but the mechanism is unknown. Notably, emerging findings indicate that mitochondrial autophagy also occurs in a Parkin-independent manner during aging,^186^ but the exact mechanism behind this phenomenon remains uncertain, thus implying the complexity of mitophagy. In addition to mitochondrial triggers, several proteins have been identified as mitophagy receptors, including FUNDC1, BNIP3, NIX/BNIP3L, Bcl2L13, FKBP8, and prohibitin-2 (PHB2), in governing mitophagy.^187–191^ However, our knowledge to the fate of these proteins in aging heart is limited, implying the importance of future work. Sirtuins are also involved in autophagy, and they directly regulate metabolic and structural remodeling in cardiac aging. For example, Sirt1 promotes autophagosome genesis and its fusion with lysosome by stimulating FoxO1 deacetylation-dependent transcriptional activation of Rab7 in the heart.^192^ In addition, an age-induced reduction in NAD^+^ results in sirtuins suppression and lysosomal dysfunction,^193^ which further disrupts autophagy-lysosome formation and the accumulation of dysfunctional mitochondria. However, excessive removal of mitochondria, including robust IFM inside the myofibrils, may lead to degradation.^194^ Understandably, the improper removal of IFM by excessive autophagy reduces IFM content with cardiac aging.^195^ Multiple mechanisms are used to eliminate damaged mitochondria, including Parkin-independent macroautophagy, mitochondrial proteases,^196^ ubiquitin proteasome-dependent degradation,^197^ and mitochondria-derived vesicles.^198^ Most recently, mitocytosis has been uncovered as a novel way to eliminate aberrant mitochondria and monitor mitochondria quality control,^199^ which underscores the need for further research since our understanding of aging-related mitocytosis remains unclear. Emerging evidence from multiple studies suggests that aging accelerates mtDNA mutation, which impedes autophagy-related impaired mitochondrial degradation by activating mTOR^200^ and cGAS-STING signaling.^201^ Currently, the effects of cardiac aging on these mechanisms have yet to be formally investigated. Although emerging evidence has confirmed the link between aging and autophagy in cardiomyocytes, the effects of autophagy upon endothelial cells, fibroblasts, and macrophages has not been fully recognized. Importantly, the outcome and importance of scrapable mitochondria is almost completely far from clear, and investigation of “the waste” is a promising avenue for future exploration and will without doubt be the subject of cardiac aging studies.

### Electron transport

**Fig. 4:**
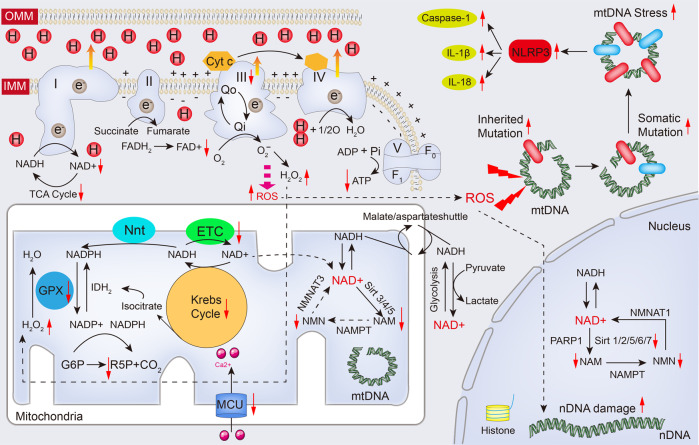
Altered NAD(H), ROS and mitochondrial DNA during cardiac aging. The electron transport chain (ETC) is a collection of four enzyme complexes (complexes I-IV) and a large protein complex (complex V) responsible for synthesizing ATP in the mitochondrial inner membrane. It generates ATP from ADP, Pi, and Mg2+ using an electrochemical gradient of protons created by the electron transport chain. As electrons pass down the redox potential gradient from NADH or FADH2 to oxygen, hydrogen ions are actively transported from the matrix to the cytosolic side of the inner membrane by complexes I, III, and IV. Complex I oxidizes NADH, which leads to sequential electron flow to coenzyme Q, complex III, cytochrome c, and ultimately to cytochrome oxidase (complex IV), where oxygen is reduced to water. However, during cardiac aging, the decline in respiration favors the relative reduction of complexes I and III, leading to increased ROS production. This increased ROS contributes to the impairment of the Krebs cycle and reduced ATP production. Furthermore, the cytoplasmic and nuclear NAD^+^ pools probably equilibrate by diffusion through the nuclear pore. However, the mitochondrial membrane is impermeable to both NAD^+^ and NADH. Reducing equivalents generated by glycolysis are transferred to the mitochondrial matrix via the malate/aspartate shuttle. In addition, different NAD^+^-consuming enzymes lead to the generation of nicotinamide, which is recycled via the NAD^+^ salvage pathway. Different forms of the NMNAT enzyme and sirtuins are localized in different compartments. Of note, a proportion of ROS is involved in reduction reaction by mitochondrial antioxidant system GPX device. While overburden of ROS leads to mtDNA mutation and damage to promote activation of NLRP3 inflammasome. Red arrows indicate alterations that occur in the aging heart (see text for details). ADP adenosine diphosphate, ATP adenosine triphosphate, Cyt c cytochrome c, FAD flavin adenine dinucleotide, FADH_2_ reduced flavin adenine dinucleotide, F_1_/F_o_ F_1_/F_o_ ATP synthase, G6P glucose-6-phosphate, GPX glutathione peroxidase, IL-1β interleukin-1β, IL-18 interleukin-18, IDH_2_ isocitrate dehydrogenase 2, IMM inner mitochondrial membrane, MCU mitochondrial Ca^2+^ uniporter, mtDNA mitochondria DNA, NADH nicotinamide adenine dinucleotide, NADPH reduced nicotinamide adenine dinucleotide phosphate, NAM nicotinamide, nDNA nuclear DNA, NLRP3 NOD-like receptor family pyrin domain containing 3, NMN nicotinamide mononucleotide, NMNAT1/3 nicotinamide nucleotide adenylyltransferase 1/3, Nnt mitochondrial NAD(P) transhydrogenase, OMM outer mitochondrial membrane, PARP1 poly(ADP-ribose) polymerase, Pi phosphate ion, ROS reactive oxygen species, R5P ribulose 5-phosphate, TCA cycle tricarboxylic acid cycle. The online resource inside this figure was quoted or modified from Servier Medical Art

The transfer of electrons in the electron transport chain (ETC) drives the generation of ATP within cells. It couples with the tricarboxylic acid (TCA) cycle and oxidative phosphorylation (OXPHOS) in mitochondria.^202^ In the electron transport chain, electrons are transferred along a redox potential gradient from NADH or FADH2 to oxygen, causing hydrogen ions to be transported from the mitochondrial matrix to the inner membrane, and multisubunit enzyme complexes, including complexes I-IV, are involved in this process^203^ (Fig. 4). In line with the pathological mechanisms underlying congestive heart failure (CHF), cardiac aging disrupts the mitochondrial respiratory of cardiomyocytes by interrupting enzyme function and content of ETC complexes and suppresses the organization of respirasomes (supercomplexes). The function of complex I is to oxidize NADH, resulting in the flow of electrons into coenzyme Q. As mentioned before, aging-related mitochondrial defects is characterized with reduced NAD^+^ content and NAD:NADH ratio, and an increased NADH content may restrain the enzyme activity of complex I.^204^ It is demonstrated that restored ability of NAD^+^ regeneration in complex I can rescue brain degenerative progression and expand the lifespan.^205^ This is in line with a prior study that suggested that enhanced complex I activity is predicted to not only directly prevent the defects in mitochondria in aging, but also significantly induce NAD^+^ accumulation in cells, thereby promoting the activation of sirtuins.^206^ These data strongly support that NAD^+^ regeneration seems to be the primary mechanism by which mitochondrial complex I acts against degeneration and aging, while the protection of complex I in cardiac aging remains elusive.

Defects in the mitochondrial inner membrane environment occur during cardiac aging and contribute to the inactivation of cytochrome oxidase (complex IV), thus restraining the suppressed reaction of oxygen in the ETC.^207^ Importantly, the content and constitution of cardiolipin in IFM have been found to support the microenvironment of cytochrome oxidase, with subsequent regulation of enzyme activity.^203,208^ In actuality, aging downregulates the elasticity of the inner membrane and the microenvironment of mitochondria due to severe oxidative damage to membrane phospholipids.^209^ Similarly, OPA1 was found to bind with complex IV and enhance its activity in mitochondria, thereby contributing to increased longevity in mice.^210^ Unexpectedly, complex IV-associated Surf-ablating mice presented defective reaction of complex IV, improved insulin response, and boosted genesis of mitochondrial, which thus prolonged the lifespan as well;^211^ however, the involvement of downregulation in extending lifespan of preclinical models needs to be assessed. The contradictory results of targeting complex IV may be attributed to distinct mechanisms, and the underlying mechanism remains unclear. Notably, how targeting complex IV impacts on cardiac aging also requires more assessment. Besides altered enzyme activity, complex IV net content is reduced with aging and accompanied by enhanced cytotoxicity and endoplasmic reticulum (ER) stress activation,^212^ both of which trigger mitochondrial ROS production. Composed of cytochrome b/c1 and the iron-sulfur protein, complex III is vulnerable to cellular senescence and functions as the factory of ubiquinol oxidation and electron transfer.^213^ Similarly, aging significantly counteracts the maximal activity of complex III inside the mitochondrial inner membrane.^214^ In addition, mutation of the ubiquinol-binding site (Qo) of cytochrome b may result in the aging phenotype of complex III, which could be utilized as novel target for drug development.^215^ For example, the mutation at Y132 contributes to the decrement in complex III activity,^216^ and promotes aging-induced oxidative modification inside mitochondria. Along with the rapid development and application of proteomic techniques, multiple omics have uncovered the distinctive mechanism inside complex III during cardiac aging. Likewise, the activity of ATP synthase [also known as complex V] is negatively correlated with aging,^217,218^ resulting in a substantial decrement in the efficiency of OXPHOS. Moreover, complex V serves as a potential locus of the mitochondrial permeability transition pore (mPTP),^219^ while defective complex V restrains the formation of mPTP and limits the coupling of OXPHOS to the ETC. Further, Angeli et al. identified mPTP as a pathological pore that contributes to aging by activating the mitochondrial unfolded protein response (UPR^mt^).^217^ Thus, the contents and enzyme activity of complexes in the ETC might play different roles in OXPHOS and energy production. However, the mechanism behind this remains unclear, and the influences of complex V on the cardiac aging requires further investigation. Currently, potential approaches for targeting the ECT in cardiac aging remain to be determined, and more research is necessary to translate this information into practical and effective strategies.

### NAD^+^ metabolism and sirtuin

**Fig. 5:**
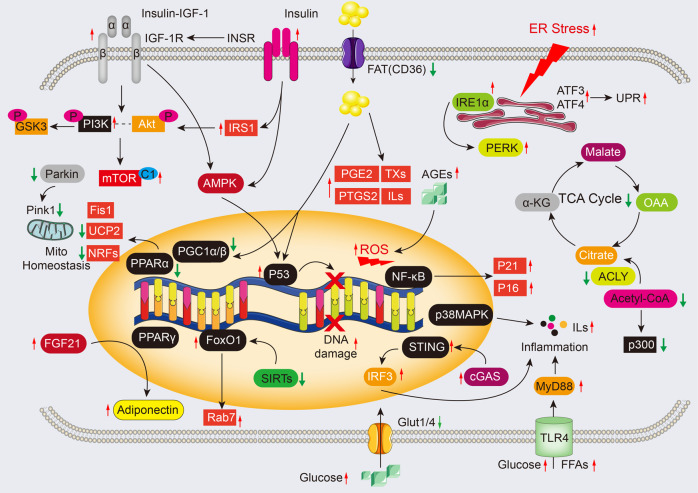
Molecular mechanisms and potential signaling for cardiac aging. The insulin/IGF-1 signaling pathway can activate signal transduction through the PI3K/Akt pathway, which in turn phosphorylates multiple targets, regulating the activity of the mTOR complex (mTORC1). IGF-1 also contributes to protein synthesis by activating the PI3K/Akt/mTOR and PI3K/Akt/GSK3β pathways, which can lead to endoplasmic reticulum (ER) stress or protein degradation via the ubiquitin-proteasome system (UPS). In the presence of ER stress, activated IRE1α and PERK can initiate proinflammatory and proapoptotic signaling pathways. Increased NAD^+^ might contribute to SASP and aging by disturbing AMPK and p53 activation, as well as enhancing p38MAPK and NF-kB activity in senescent cells. During cardiac aging, the maintenance of mitochondrial homeostasis is impaired, which can result in the release of mtDNA and the activation of the cGAS/STING/IRF3 pathway, leading to the production of inflammatory cytokines. The PINK1-Parkin pathway plays a crucial role in maintaining mitochondrial function and dynamics through mitophagy. In addition, ROS can activate the MAPK and PI3K/Akt signaling pathways, as well as increase levels of p53 and p21, which can promote apoptosis and inflammation in chondrocytes. The TLR4-MyD88 pathway can also be activated by circulating FFAs and glucose, leading to the production of proinflammatory factors. The online resource inside this figure was quoted or modified from Servier Medical Art

Compared with young hearts, adult and aged hearts have much lower nicotinamide adenine dinucleotide (NAD^+^) levels and higher NADH levels, with therefore lowered cytosolic NAD^+^/NADH ratios.^220^ In addition, sirtuin and poly-ADP-ribose polymerase (PARP) is reduced in aged hearts. Being a critical cofactor for proteins supporting reduction-oxidation (redox) reactions, NAD^+^ is widely distributed in various cells and transmits electrons from one reaction to another.^221^ The decrease in mitochondrial function is accompanied by reduced NAD^+^ amount and the NAD:NADH ratio, both of which compromise the capabilities of NAD^+^-dependent proteins, including sirtuin and PARP. Notably, NAD^+^ pools decline during aging,^222^ and the decrement in NAD^+^ balance may also be observed in nearly diseases associated with age, including aging hearts.^223^ NAD^+^ levels inside cells are very much dictated by the match between de novo synthesis and tryptophan in kynurenine pathway-mediated salvage pathways and consumption by sirtuins and PARP^224^ (Fig. 4). Notably, PARP has been verified to counteract cell senescence by restraining genotoxic stress,^225^ while PARP also contributes to proinflammation and the SASP phenotype in senescent cells,^226^ implying the distinct effects of PARP on senescence initiation and established aging. Blockade of PARP1 antagonizes cell cycle arrest and defective DNA repair by increasing p38MAPK levels in senescent cells.^227^ More importantly, targeting PARP attenuates aging-associated cardiac and vascular dysfunction by improving mitochondrial function and communication.^228,229^ Conversely, unlike PARP, sirtuin family proteins (SIRTs) play negative roles in the proinflammatory SASP and senescence response. For example, Sirt 1 is downregulated by autophagy in senescence and aging,^230^ but Sirt 1 deletion triggers several degenerative alterations in atherosclerosis, neurodegeneration, and cirrhosis by increasing the SASP and cell cycle arrest.^231,232^ In addition, Sirt 6 might serve as a safeguard against the initiation of aging-related senescent myocyte and cardiac hypertrophy,^233–235^ particularly exercise-associated protection in the aging heart.^236^ However, Sirt 6-deficient mice showed premature aging and presented a proinflammatory phenotype.^237,238^ Both Sirt 1 and Sirt 6 have been found to prevent cardiac aging.^239,240^ Moreover, Sirt 2 protects against cell senescence and aging by restoring BUBR1, the mitotic checkpoint kinase, and H3K18Ac activation.^241,242^ Although a previous study confirmed the anti-cardiac hypertrophy effect of Sirt 2,^243^ whether Sirt 2 regulates cardiac aging is not clear. In line with PARP, elevated NAD^+^ may present the contrary influences and contribute to the SASP and aging by disturbing AMPK and p53 activation as well as enhancing p38MAPK and NF-kB activity in senescent cells^244,245^ (Fig. 5). These and many other studies suggest that NAD^+^ may play different roles at different times in aging. Nicotinamide phosphoribo-syltransferase (Nampt) is the enzyme that catalyzes the initial step in the NAD^+^ salvage pathway in mammals, which is rate-limiting.^246^ NAD^+^ synergizes with sirtuin to regulate protein acetylation and contributes to the improvements in DNA damage and oxidative stress in aging hearts, while downregulated NAD^+^ with defective Nampt aggravates this cardioprotection.^221^ Emerging evidence has shown that Nampt is substantially reduced in the ischemic heart, inducing a decrease in NAD^+^ content, inhibition of autophagic flux, and loss of cardiomyocytes.^247^ In contrast, increased NAD^+^ levels through Nampt overexpression led to the induction of autophagy during ischemia and an improvement in cardiac injury in myocardial infarction.^193,248^ In terms of the mechanism, a decrease in NAD^+^ levels promote lysosomal dysfunction, increasing the aging heart’s vulnerability to ischemic injury.^249^ These lines of evidence highlight essential roles for NAD^+^ in redox maintenance and suggest that replenishing NAD^+^ with NAD^+^ precursors, including nicotinamide and nicotinamide riboside, may have broad benefits for both lifespan and quality of health (Fig. 4).

### Mitochondrial ROS and mtDNA

Increased ROS emission is an early warning sign for multiple pathological cardiac phenotypes. Mitochondria, especially the quinol oxidation site (Qo center) at complex III, serve as the major site for ROS release,^250^ in which enhanced ROS production appears before the functional alteration (Fig. 4). Interestingly, the mitochondria-ER interaction sites are also closely associated with the complex III Qo site that generates mitochondria ROS,^251^ by which ER stress may produce a rise in ROS^252^ and trigger mitochondrial ROS-indued ROS release.^253^ In terms of the potential mechanisms, reducing respirasomes decreases the redox hubs that straightly respond to O_2_ to produce ROS during cardiac aging. In addition, the activity and content of individual ETC complexes are substantially reduced in cardiac aging, directly favoring ROS assembly. Moreover, defective complex III inside the IFM instead of the SSM is a contributor to the elevated ROS assembly in aging hearts; consistent with the enhanced ROS assembly in IFM, markers of oxidative stress are upregulated in these cellular compartments during aging.^254,255^ Redox metabolism inside mitochondria serves as a crucial signal for cell fate. The combination of metabolite oxidation and oxidant production within mitochondria is crucial for viable cardiomyocytes during cardiac aging. For example, nuclear factor-erythroid 2-related factor 2 (NRF2) serves as a redox-responsive factor and mediates ROS elimination,^256^ while downregulation of NRF2 is observed in elderly individuals. As expected, reduced ROS production or the oxidized form of glutathione (GSSG) disposition ameliorates cardiac aging,^257,258^ supporting the concept that ROS derived by mitochondria are harmful to aging hearts. Recently, the correlation between ROS with cardiac aging is reported in some preclinical studies. Ablation of mitochondrial superoxide dismutase (SOD2), a ROS scavenger, leads to the development of senescence and aging.^259^ SOD3 mutation drives ROS-mediated chronic inflammation and degenerative diseases in aged mice.^260^ Furthermore, mitochondrial ROS triggers the Jun N-terminal kinase, thereby inducing the secretion of chromatin fragments from cytosol and enhances SASP.^261^ Several studies have suggested the possible preservative effects of limited assembly of ROS with classic ischemic preconditioning.^262^ However, the notion of ischemic preconditioning protection does not apply to elderly patients due to excessive ROS content during aging. Generally, it is an open question whether the promoted senescence due to aging is attributed to mitochondrial ROS. Notably, ROS actually serve as key molecules for several cardio-pathological processes, including cardiomyocyte renewal,^263^ fibroblast proliferation,^264,265^ differentiation,^266,267^ immune response^268^, and cardiomyocyte survival,^269^ which highlights the complex relationship between ROS and cardiac aging, and requires further investigation.

Due to the absence of protective histones, mtDNA is highly susceptible to oxidative damage.^270^ In mammalian species, accumulated mtDNA mutations have also been reported to contribute to aging,^271^ disturbing mitochondrial metabolism and resulting in dysfunctional consequences for targeted organs (Fig. 4). The chance of mutations on mtDNA of aged mice is approximately 1000-fold higher when compared with nuclear genes.^272,273^ A substantial portion of proteins critical for the genesis and capability of mitochondrial respiratory complexes, particularly complex III inside mitochondria, are determined by mtDNA, in addition, the mtDNA copy number is crucial for mitochondrial function.^274^ Elevation in oxidative stress contributes to the suppressed mtDNA replication and decrements in mtDNA copy number, causing mitochondrial respiratory chain deficiency and aging-related impairment of metabolism.^275^ Importantly, increased ROS content drives cardiomyocyte cell cycle arrest through mtDNA damage.^276^ In addition, defective mtDNA excision repair can lead to the accumulation of point mutations,^277^ double-strand breaks and, eventually, large deletions, all of which are associated with cardiac aging and dysfunction. For example, a recent study reported that a POLG-mutant mouse carrying a D257A mutation in a key residue of mitochondrial DNA polymerase experienced a loss of mtDNA stability, leading to a significant acceleration of aging processes.^278,279^ Moreover, increased mtDNA mutation burden contributes to abnormal mitochondrial biogenesis resulting in programmed cell death in cardiomyocytes^280,281^ (Fig. 4). Recently, studies have revealed that the secretion of mtDNA from the mitochondria, into the cytoplasm, and ultimately into the extracellular milieu accelerates aging and degenerative alteration by enhancing inflammation, innate immune signaling, and programmed cell death in local tissues.^282,283^ In addition, the extracellular release of mtDNA is emerging as intercellular signaling to mediate cell-to-cell crosstalk in the pathogenesis of CVDs.^284^ Collectively, both accumulated ROS production and increased mtDNA mutation burden may serve as senescent pioneers before functional alteration in cardiac aging. Of note, the extracellular release of mtDNA needs to be further investigated in future studies.

## Therapeutic implications

As outlined above, these results demonstrate the intricate link between metabolism and cardiac aging, therefore prompting us to explore interventions that target metabolism that might serve as an approach for cardiac aging or delay aging progression. Five drugs, including rapamycin, acarbose, nordihydroguaiaretic acid, 17-α-estradiol, and aspirin, have been recognized by the multicenter Intervention Testing Program (ITP) supported by the National Institute for Ageing, as capable of reproducibly increasing lifespan in mice.^285^ Below, we outlined the possible interventions targeting metabolism in aging hearts and identify future challenges in this field.

### Targeting metabolites and intermediates

Although clear evidence of substantially defective fatty acid (FA) oxidation is emerging as a crucial trigger for metabolic remodeling and dysfunction in cardiac aging, drugs targeting metabolites are not routinely utilized in the clinical field. Since glycolysis requires less oxygen than FA oxidation as a consequence of the same amount of ATP,^286,287^ blockage of FA utilization may benefit cardiac bioenergetics under normal glucose uptake and utilization conditions. However, insulin resistance antagonizes hepatic glucose output and reduces the glucose uptake in cardiac aging, thereby defying this assumption. In addition, some toxic intermediates of FA oxidation further accelerate the accumulation of lipid droplets in heart tissues during aging and restrain cardiomyocyte survival. Thus, supplementation with FA with highly efficient utilization may benefit the aging heart. Accordingly, omega-3 fatty acids are emerging as a major constituent of the cell membrane used for restriction of age-correlated disease on account of considerable epidemiological evidence, such as the Age-Related Disease Study 2 (AREDS2).^288,289^ Omega-3 fatty acids reduce triglyceride amounts and bind with enzymes required for the biosynthesis of lipid mediators.^290^ Likewise, an omega-3 fatty acid diet may counteract cardiac aging.^291,292^ Moreover, recommendations for the dietary consumption of omega-6 polyunsaturated fatty acids (PUFAs) support CVDs prevention,^293^ which further suggests that the omega-6 PUFA diet may improve cardiac dysfunction in elderly individuals. Thus, enhancing the FA utilization rate with a linoleic acid (LA) supplement is a possible method for slowing the impairment of cardiac aging (Fig. 5). In addition, selective PPARα agonists (fibrates) decrease myocardial FA supply and uptake by promoting their utilization in extracardiac tissues and impeding lipid disposition in heart tissues.^294^

**Fig. 6:**
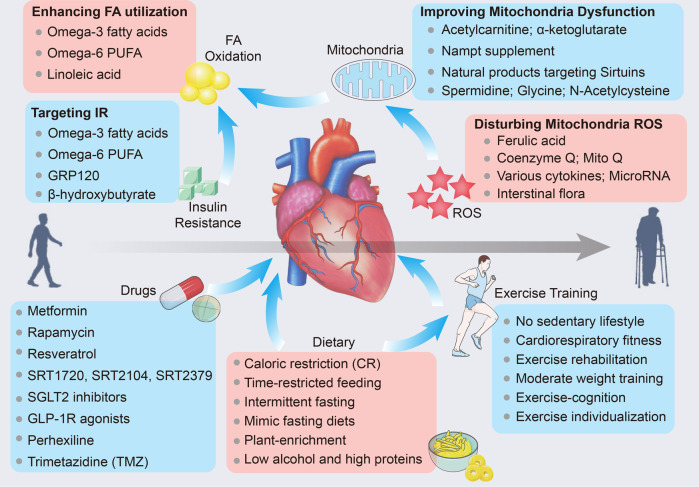
Potential therapeutic approach for cardiac aging. Metabolic therapies for the treatment of cardiac aging are aimed to improve insulin resistance, FA oxidation, mitochondria dysfunction, and ROS. Synthetic small molecular drugs targeting insulin resistance, sirtuin activation, and ROS clearance are verified to delay cardiac aging in animal model. Moreover, both dietary advice and exercise training are beneficial for cardiac aging in the elderly. The online resource inside this figure was quoted or modified from Servier Medical Art

In addition, targeting insulin resistance and glucose oxidation may serve as alternative therapeutic strategies to ameliorate metabolic remodeling and improve cardiac efficiency during cardiac aging (Fig. 6). Likewise, omega-3 and omega-6 fatty acids have been reported to reduce inflammation and insulin resistance.^295,296^ GRP120 is recognized as an omega-3 fatty acid receptor that ameliorates inflammation and optimizes potent insulin sensitization.^297^ This evidence further favors the hypothesis that omega-3 and omega-6 fatty acid supplements may improve aging hearts. In addition, optimizing glucose oxidation with dichloroacetate stimulates glucose utilization by inhibiting its phosphorylation by promoting pyruvate dehydrogenase (PDH) activity.^27^ Ketone bodies, especially β-hydroxybutyrate (β-HB), are sourced from FA oxidation and possibly act as an energy origin for heart failure; accordingly, β-HB has the potential to be utilized as ancillary therapy for cardiac aging.^102^ Moreover, β-HB suppresses mitochondrial dysfunction by disrupting NLPR3 inflammasome formation and antagonizing the proinflammatory SASP in aged mice.^298^ In addition, both the pan carnitine O-palmitoyl-transferase 1 (CPT1) inhibitor perhexiline^299,300^ and the acetyl-CoA acyltransferase 2 inhibitor trimetazidine (TMZ)^301,302^ are metabolic reprogramming modulators that have the ability to partially suppress mitochondrial free FA beta-oxidation partially, enhance glucose oxidation, and promote cardiac energetics (with an increased PCr:ATP ratio). The latter has been further suggested to halt cardiac aging as well as metabolic defects in animal models,^303,304^ but there is a lack of clinical evidence and a very limited number of patient studies. Most recently, Na^+^/glucose cotransporter 2 (SGLT2; also known as SLC5A2) inhibitors have been verified to improve cardiac metabolism in type 2 diabetes mellitus,^305,306^ and SGLT2 inhibitors have been found to delay vascular aging by improving vascular function.^307^ Further, SGLT2 inhibitors impede the assembly of the more oxygen-efficient substrate β-hydroxybutyrate, a kind of ketone body, in the heart.^308^ A preclinical study reported that malfunctional SGLT2 in cardiac aging mediates defects in mitochondrial and cardiac contractility.^309^ Likewise, glucagon-like peptide 1 receptor (GLP-1R) agonists are also ascertained to reverse aging and neurodegeneration at a genetic level.^310^ However, their role and precise mechanism in cardiac aging remain to be further explored. Moreover, targeting sirtuins such as Sirt1 is significant in slowing cardiovascular aging,^311^ and recently, sirtuin-activating compounds, including resveratrol, SRT1720, SRT2104, and SRT2379, are also made to trigger the action of sirtuin to mitigate certain age-associated conditions in rodents and nonhuman primates^231,312^ (Fig. 6). Specifically, various small-scale clinical investigations on the impacts of SRT2104 on cardiovascular and metabolic markers, including those in type 2 diabetes patients, cigarette smokers, and elderly individuals,^313^ have been completed, with larger trials underway. Notably, the mismatch between FA oxidation and uptake seems to be opposite to insulin resistance and reduced energy fueling during cardiac aging. Cardiovascular aging-induced metabolite utilization seems to have conflicting outcomes, and targeting single metabolic pathways would be more straightforward.

### Improving mitochondrial dysfunction

As outlined above, enhancing mitophagy, optimizing electron transport, and blocking ROS and mtDNA mutations have emerged as potential strategies to improve mitochondrial dysfunction during aging. Among these, suppression of mitochondrial ROS production has always been a focus, while the past few decades have seen enormous strides to target mitochondrial ROS content. Ferulic acid, a powerful natural antioxidant and scavenger of free radicals, occurs naturally and is well recognized for its beneficial properties; it contributes to lifespan and stress resistance by interrupting ROS accumulation.^314^ Acetylcarnitine, a tracer of acetyl-CoA, optimizes aging-mediated reduction in OXPHOS, complex III, and complex IV by stimulating the transcription of mtDNA linked to ETC subunits.^315^ Metformin correlates with an prolonged lifespan in patients by promoting mitochondrial respiration. Accordingly, it was the initial drug assessed for its age-targeting influences in the large clinical study TAME.^316–318^ Furthermore, a retrospective analysis of patients with diabetes who received metformin showed a prolonged lifespan compared with individuals without DM.^319^ Importantly, as one of the most crucial antioxidants, coenzyme Q (CoQ) functions as an electron acceptor that obtains electrons from ROS (ROS scavenger) within the mitochondrial respiratory chain.^320^ It is also an essential cofactor in OXPHOS.^321^ Levels of CoQ in plasma are substantially reduced in HF patients,^322^ and CoQ content essentially declines with age.^323^ According to the Q-SYMBIO trial, continued CoQ10 supplementation to HF patients shows beneficial impacts on HF symptoms, reduces major adverse cardiovascular events (MACEs), and serves as the adjunctive treatment in chronic HF.^324,325^ Consistently, in several preclinical studies, CoQ has been suggested to improve aging-induced mitochondrial dysfunction by counteracting oxidative stress in the heart.^326^ Therefore, CoQ supplementation attenuates cardiac fibrosis and aging,^327^ with favorable clinical applications in CVDs. Subsequently, an optimized Mitoquinol mesylate (MitoQ), the combination of CoQ and lipophilic cation TPP^+^ is generated, and it is more likely to be absorbed by mitochondria than CoQ.^328^ Various animal models have verified the beneficial effects of MitoQ upon HF and aging.^329,330^ However, the evidence of MitoQ application in patients is limited, and consistently the roles of CoQ and MitoQ in cardiac aging need further investigation. Otherwise, various cytokines, miRNAs, and exosomes from the heart or distal organs also determine ROS during cardiac aging, which may be potential strategies (Fig. 6).

In addition to ROS production, suppressing mTOR with rapamycin is found to alleviate the unfavorable effects of cardiac aging and increase lifespan by promoting autophagy.^331^ In addition, everolimus, an analog of rapamycin has been authorized in clinical utilization as an immunosuppressant in transplanting solid organs, and importantly, healthy older individuals who received a non-immunosuppressive dose of everolimus presented an improved immunological response;^332^ however, observations mentioned above is limited by concentrating on influences observed 6 weeks after everolimus administration and chronic exposure was not considered. In the following year, a preclinical study revealed that chronic mTOR inhibition prolongs the life of immune-deficient mice by modestly altering gut metagenomes, and some metagenomic impacts correlated with immune outcomes.^333^ Unfortunately, the mechanism behind this remains unclear and mTOR inhibitor immune effects merit further studies associated with prolonged lifespan. As expected, proper amounts of exercise (discussed blow) can delay cardiac aging by enhancing autophagy and stimulating the phosphorylation of Bcl-2 and its dissociation from Beclin1.^334^ Enhanced de novo NAD^+^ synthesis, exogenous Nampt supplementation, or sirtuin targeting may promote NAD metabolism in mitochondria, serving as a potential approach in cardiac aging.^335^ Most recently, alginate oligosaccharide (AOS) was identified as an effective agent in impeding cardiac aging by improving mitochondrial biogenesis and maintaining mitochondrial integrity in aged mice.^336^ Similarly, the mitochondrially targeted peptide elamipretide (SS-31) significantly alleviated mitochondrial ROS and protein oxidation in aged hearts by targeting cardiolipin.^337^ Senolytic drugs that target and eliminate senescent cells present great potential in aging, all of which are involved in natural products and synthesize small molecules^338,339^ (Fig. 6). Likewise, there is insufficient clinical evidence supporting the use of these strategies in cardiac aging.

### Healthy lifestyle for a healthy heart—dietary and exercise interventions

The Western diet is one of the main reasons for the growing obesity epidemic, chronic diseases, and aging. There is strong evidence that eating habits such as dietary restriction, overeating, and the resulting obesity influence both the lifespan and quality of health.^340^ Caloric restriction (CR) delays cardiac aging by improving mitochondrial bioenergetics by reducing oxidative stress and activating sirtuins.^341^ Optimal dietary practices that support longevity and health encompass a diet rich in plant-based foods, few processed foods, low alcohol consumption, and high protein consumption.^340^ Emerging evidence in the nutrition field is presented to promote health and lifespan, such as time-restricted feeding,^342^ intermittent fasting^343^, and diets that mimic fasting.^344^ Most recently, a ketogenic diet with high amount of the ketone body β-hydroxybutyrate is verified to increase lifespan and health.^94^ Future research should focus on delaying cardiac aging through the dietary strategies and identifying their correlations with precise mechanisms that modulate cardiac aging (Fig. 6).

Maximal aerobic exercise capacity decreases throughout adulthood and accelerates in later years. In addition to optimal diet and drug development, proper exercise is an effective protector for reducing the incidence of age-related disease.^345^ Overwhelming evidence supports the importance of maintaining high intensity of physical activity, engaging in exercise training, and improving overall cardiorespiratory fitness as effective strategies for treating CVDs.^346^ Mechanistically, a moderate amount of exercise activates sirtuin-1 and sirtuin-3,^347,348^ synchronously decreases mitochondrial production of H_2_O_2_ with increased MnSOD activity,^348^ and supports OXPHOS and mitochondrial metabolism, antagonizing fibrosis and proapoptotic signaling in the aging heart.^349^ Currently, despite the beneficial influences of exercise against aging, the mechanisms and whether this knowledge can be utilized to enhance the hearts of the aging population need further investigation.

## Future challenges

### What initiates senescence and cardiac aging?

Research advances have linked potential interventions with cardiac aging, whereas the identification of biomarkers for assessing the aging process remain a challenge. Our understanding of the in vivo processes responsible for aging and the induction of senescent cells, particularly in the context of cardiac aging, remains limited. Metabolic warning signs usually occur before cardiac structural alteration and dysfunction, providing a potential approach to detecting metabolic biomarkers during cardiac aging. Although cardiac aging is involved in metabolic disarrangement, DNA damage, telomere attrition, and mitochondrial morphofunctional defects, it has been difficult to determine how they induce senescent cells in the heart. Likewise, the development of multiple omics techniques, including single-cell and spatial omics, seems to provide a spectrum of choices for biomarkers in cardiac aging. Notably, long term and continuous observation in the elderly individuals is required to identify persuasive biomarkers of cardiac aging.

### How can cardiac aging progression be delayed?

In addition to targeting metabolites in cardiac aging as described above, the modulation of mtDNA replication and stability can improve mitochondrial metabolism and energy fueling by promoting mtDNA-encoded catalytic subunits of complexes inside mitochondria, which may offer a novel concept for cardiac aging intervention with the tremendous progression of gene editing therapy and CAR T cells.^350–352^ Furthermore, telomerase activity is typically reduced as an organism age due to increased ROS production in senescent cells. This phenomenon has been implicated in cardiac aging,^353^ and restoring telomerase activity seems to be a potential approach. Recently, mitochondrial telomerase improves mitochondrial complex I subunit composition, which is responsible for cardioprotection in ischemic cardiomyopathy.^354^ In line with a previous study, telomerase defects correlate with the deterioration of heart tissue repair.^355^ Although the link between cardiac pathology and telomerase has been verified over the past decade, there are insufficient clinical data to support its translational value. Importantly, the mechanistic exploration of cardiac aging is relatively superficial in preclinical research, and more therapeutic targets for cardiac aging require further investigation in basic research. Specifically, to accelerate the development of precision medicine for delaying cardiac aging, it is essential to promote greater collaboration among researchers, doctors, patients, and data systems, among other stakeholders.

### How can we move from simple organisms to humans?

Although mice are commonly used for research, it is widely acknowledged that essential outcomes observed in mice do not always translate to humans. Although there are many examples of a connection between cardiac aging and the treatment of CVDs, an important consideration is the methodology for testing these interventions and their eventual clinical application in human populations. First, several interventions that are beneficial in a specific genetic scenario may not apply to another.^356^ That said, the natural genetic variations present in the population may dampen the curative effects of pharmacological intervention, as explained above. Precision medicine has great potential to discover crucial genetic players of aging and to customize strategies to unique genetic variants, because of the large genetic heterogeneity in the human population. Second, despite numerous animal studies suggesting the benefits of nutrition and exercise, some research has challenged the universal applicability of these interventions. Both exercise and dietary restriction have arisen from preclinical studies to present protection against aging in animals,^357–359^ while it is plausible that individuals who have already optimized their nutrition and exercise habits may not derive significant benefits from further interventions. Subsequently, the same management for aging result in distinct outcomes due to gender differences, even in human investigations. Last, both the intricate nature of biology and the diverse range of biological phenotypes lead to reproducibility problems between different investigators, not only in mouse research but also in other model systems, implying that findings from studies conducted in mice do not always accurately predict outcomes in humans. Thus, more clinical trials are expected to provided more convincing clinical evidence instead of animal experiment in the future.

## Conclusions

The aged heart exhibits accumulated ROS and lipids, with concomitant cardiac hypertrophy and diffuse fibrosis, therefore resulting in cardiac remodeling and dysfunction. The rapid increase in our understanding of mitochondrial metabolism that underlies cardiac aging contributes to making recommendations to intervene in aging-related cardiac complications. Importantly, the heart requires continuous energy fueling and relies predominantly on mitochondrial OXPHOS, while defective metabolism and abnormal mitochondria are part of the pathophysiology of cardiac aging prior to heart failure and concomitant clinical symptoms. Cardiac aging initiates shifts in substrate oxidation with impairment of FA oxidation and dysregulated glucose utilization, along with lipid storage and ROS generation in the heart. Improper substrate utilization and increased oxygen radicals are thought to lead to defects to mitochondria, damages to adjacent organelles, and cell death in the elderly heart. Accumulated clues to understanding cardiac aging present a chance to explore novel strategies which could be beneficial to cardiac regenerative diseases, particularly metabolic remodeling, which may serve as an early warning sign in elderly patients. At present, several interventions targeting mitochondria and/or metabolism have already been suggested to delay the development of cardiac aging, including inactivation of ROS with antioxidants, inspiration of mitophagy, Nampt supplementation, and stimulation of sirtuins. In addition, emerging developments in the nutrition field including CR diets, intermittent fasting, and ketogenic diets, have been recently shown to benefit the aged heart. However, before considering the translation of these interventions for the treatment of human patients, future studies should investigate their effects on both lifespan and cardiac aging.
